# Truss for the Permanent Cure of Hernia

**Published:** 1834

**Authors:** 


### IV.—Truss for the Permanent Cure of Hernia.

We have lately been shown the model of a truss invented by Thos. Stagner of Kentucky, which, according to the testimony of several medical gentlemen, is adequate to the radical cure of Hernia. It acts, of course, by pressure. Inflammation is said to be thus excited, in the degree favorable to adhesion. We have not had an opportunity of witnessing its effects. A medical gentleman of Kentucky, Dr. Keith, will spend some time in Cincinnati this summer and autumn, to render assistance to those who may choose to apply.

